# Two cases of "cannabis acute psychosis" following the administration of oral cannabis

**DOI:** 10.1186/1471-244X-5-17

**Published:** 2005-04-01

**Authors:** Bernard Favrat, Annick Ménétrey, Marc Augsburger, Laura E Rothuizen, Monique Appenzeller, Thierry Buclin, Marie Pin, Patrice Mangin, Christian Giroud

**Affiliations:** 1 Unité de Médecine du Trafic, Institut Universitaire de Médecine Légale (IUML), 1005 Lausanne, Switzerland; 2 Laboratoire de Toxicologie et Chimie Forensiques (LTCF), Institut Universitaire de Médecine Légale (IUML), Rue du Bugnon 21, 1005 Lausanne, Switzerland; 3 Division de pharmacologie et toxicologie cliniques, CHUV, 1011 Lausanne, Switzerland

## Abstract

**Background:**

Cannabis is the most commonly used illegal drug and its therapeutic aspects have a growing interest. Short-term psychotic reactions have been described but not clearly with synthetic oral THC, especially in occasional users.

**Case presentations:**

We report two cases of healthy subjects who were occasional but regular cannabis users without psychiatric history who developed transient psychotic symptoms (depersonalization, paranoid feelings and derealisation) following oral administration of cannabis. In contrast to most other case reports where circumstances and blood concentrations are unknown, the two cases reported here happened under experimental conditions with all subjects negative for cannabis, opiates, amphetamines, cocaine, benzodiazepines and alcohol, and therefore the ingested dose, the time-events of effects on behavior and performance as well as the cannabinoid blood levels were documented.

**Conclusion:**

While the oral route of administration achieves only limited blood concentrations, significant psychotic reactions may occur.

## Background

As several countries in Europe have taken policies to decrease the penalties for cannabis possession, many people especially young persons have interpreted this move as giving support to consider cannabis as a benign drug [1].

However as stated by several reports cannabis is not a harmless substance and requires urgent attention considering public health issues such as car driving for example [2]. The relationship between Cannabis and acute psychosis is another important issue. In Pakistan and also India, Bhang, a beverage made from an infusion of cannabis leaves, and flowering tops combined with milk and nuts is reported to frequently induce psychotic manifestations among consumers [3]. Presenting symptoms include grandiosity, excitement, hostility, uncooperativeness, disorientation, hallucinatory behaviour and unusual thought content [3].

Recently five large-scale longitudinal studies and a systematic review have shown that cannabis use in adolescence is associated with a two-to threefold increase in the relative risk of later developing schizophrenia [4]. Furthermore short-term psychotic reactions, particularly in naive users have also been reported. Thomas [5] describes that one in seven people reported psychotic-like symptoms. Such reactions are usually acute, transient, self-limited however very unpleasant ("hearing voices, becoming convinced that someone is trying to harm you or that you are persecuted") [6]. But cannabinoids are considered able to trigger long-lasting psychotic decompensations in predisposed individuals, which may in part account for the epidemiological association described between cannabis consumption and psychotic disorders [7,8].

The therapeutic aspects of cannabis represent additional issues, as they are in constant development since several years. Synthetic THC (dronabinol) is available for restricted medical use in the USA since 1985. Nabilone, a synthetic THC analogue, is licensed in UK for the treatment of nausea and vomiting caused by chemotherapy unresponsive to usual anti-emetics. Clinical applications actually include nausea and vomiting, muscle spasticity in demyelinating diseases, loss of appetite in cancer and AIDS, pain, insomnia, asthma as well as other applications [9].

As these oral medications are becoming increasingly available, we think it is useful to report two cases of severe psychological sides effects, especially considering the lack of data in the literature on psychotic symptoms associated with oral synthetic or natural THC.

## Case reports

We report two cases out of 8 healthy male volunteers who were included in a double blind crossover clinical study, approved by the ethics committee of the Department of Internal Medicine of the University of Lausanne. All subjects had to be occasional but regular cannabis users. Their urines were controlled to be negative for any drug of abuse (cannabis, opiates, amphetamines, cocaine, benzodiazepines) before each study period. The presence of ethanol was checked using a breathalyzer. All of them provided their written informed consent. This study was carried out to assess the effects of delta-9-tetrahydrocannabinol (THC) on psychomotor function and driving performance. It compared a medication containing 20 mg dronabinol (Marinol^R^), and 2 hemp milk decoction containing either a medium (15.8 mg average dose determination) or a high dose of THC (45.7 mg) with matched placebos. The hemp plant fragments containing 1.5 % THC and 4.4 % THC-A were provided by Hiscia institute in Arlesheim, Switzerland. After administration, blood was sampled at regular intervals for cannabinoids determination by gas chromatography coupled with mass spectrometry (GC-MS-NCI). Clinical observations and 2 psychometric tests (roadsign recognition speed and accuracy on a tracking task) were also carried out. Furthermore, the subjects were asked to report their willingness to drive and the subjective effects on a VAS scale extending from 0 to 10 cm. The effects were assessed against placebo.

These 2 cases were withdrawn from the study because of adverse events. We consider them worth reporting for the following reasons: in contrast to most other case reports where circumstances and blood concentrations are not known, our two cases reported here happened under defined clinical setting: the ingested dose, the time-events of effects on behavior and performance as well as the cannabinoid blood levels are fully documented. In addition, the consumption of other psychotropic major drugs could be ruled out.

### Case 1

The first subject was a 22-year-old medical student (weight: 65.3 kg, height: 1.82 m) and occasional cannabis smoker (about once per week). One hour after the administration of 20 mg of dronabinol, he started to laugh a lot and after 90 minutes, he manifested a severe anxiety with symptoms of derealisation and depersonalization. He reported "watching himself lying on the bed" and repeated several times the same questions at just a few minutes interval. Starting 2.5 hours after ingestion of dronabinol, and at the 4 hours and 5.5 hours post-ingestion series of tests, he was unable to perform the psychomotor tasks, despite reporting of weakening of symptoms approximately 165 minutes after their initiation. Before going to sleep (more than 10 hours after ingestion), he again felt a transient feeling of irrational anxiety and loosing the perception of his body. The next day he was well but a bit tired.

**Figure 1:**
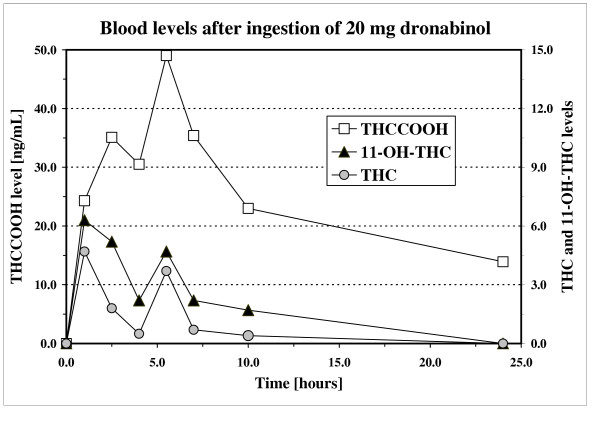
Whole blood concentrations of THC, 11-OH-THC (actives metabolites) and THC-COOH (inactive metabolite) after oral intake of 20 mg dronabinol and of a hemp milk decoction containing traces of cannabinoids (placebo).

**Figure 2:**
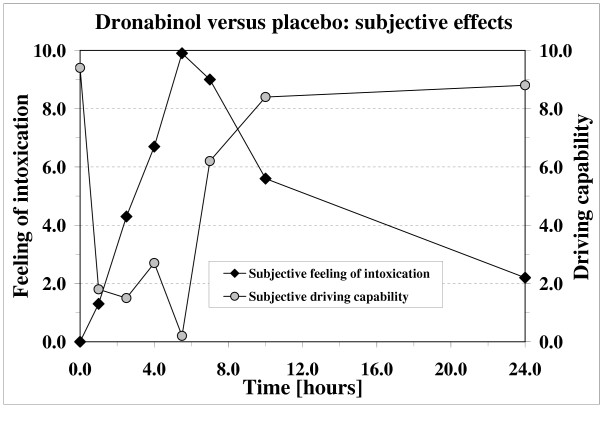
Subjective effects (feeling of intoxication or driving capability) after oral intake of 20 mg dronabinol. The subject reported no feeling of intoxication or of driving impairment after ingestion of the placebo.

Figure 1 shows the evolution of his blood concentrations of cannabinoids after ingestion of 20 mg dronabinol. At the time of strong adverse effects, the blood levels of THC and 11-OH-THC reached a concentration of 1.8 and 5.2 ng/mL, respectively. The subject reported a strong feeling of intoxication (figure 2). He also evaluated that his driving capability was strongly impaired (figure 2).

### Case 2

A 22-year-old student, also an occasional cannabis smoker (about twice a month), felt paranoid delusions with severe anxiety one hour after the administration of 16.5 mg of a THC decoction, and became suspicious during the experiment. He thought the investigators were concealing some problems. He was unable to perform the psychometric tests (roadsign recognition speed and accuracy on a tracking task) at the 1 hour and 2.5 hours post-ingestion series of tests. These effects persisted up to 4 hours after ingestion and weakened over the next 3 hours. The feeling was very unpleasant in comparison with that experienced after his usual smoking cannabis consumption.

On the next day, he was well, with no recurrence. The time-concentration curves for the major cannabinoids were similar to those observed after ingestion of 20 mg dronabinol. One hour after drinking the hemp decoction, the THC and 11-OH-THC blood levels were of 6.2 and 3.9 ng/mL, respectively. Similarly to the other volunteer, he also indicated a strong feeling of intoxication and a very important decrease in his self-reported capacity to drive (results not shown).

## Conclusion

A temporary form of drug-induced psychotic reaction after administration of oral cannabis has occurred in these two cases. Cannabis psychosis is the term proposed in the literature [10]. In 1958, Ames [11] reported in an experimental design with 10 subjects psychological symptoms such as severe anxiety, panic attacks, paranoid delusions and depersonalization. Talbott [12] in 1969 described 12 soldiers in Vietnam who had disorientation and hallucinations after their first use of cannabis. In Germany, 19 cases of toxic psychosis were reported after hashish use [13] and in Calcutta, Chopra [14] described retrospectively 200 patients hospitalized after the ingestion of large dose of cannabis between 1963 and 1968. Other reports in different countries showed similar features after bhang ingestion [3,15]. It usually results from taking large amount of the drug, generally in food or drink. The symptoms have some similarity with paranoid schizophrenia, which could raise the hypothesis that " symptoms of schizophrenic illness might be caused by an abnormal over-activity of endogenous cannabinoid mechanism in the brain" (Iversen [10] citing Emrich [16]). However because of the poor quality of information on previous cannabis experience, cannabis dose intake, other drug consumption and previous psychiatric comorbidity, some commentators have criticized these case series [12,17,18]. Case-control studies have been conducted comparing people with cannabis psychosis with persons suffering from schizophrenia [19-21]. However the results were inconsistent due in part to the small sample size of these studies.

The originality of our two cases is that they were observed in an experimental setting, and therefore adds more evidence for the ability of oral cannabis to produce psychotic symptoms. In both our subjects, the effects appeared 1 hour to 1.5 hours after oral drug intake and lasted for 3 to 4 hours. Dronabinol (synthetic THC) is reported to have an onset of action at approximately 0.5 to 1 hour and peak effects between 2 and 4 hours. Psychoactive effects last 4 to 6 hours but the appetite stimulant effect may continue for 24 hours [22].

The issue of THC dose level is very important in terms of public health. A traditional cigarette of herbal cannabis in the 1960s and 1970s contained 1–3% THC: for a joint made of 750 mg of cannabis plant, the corresponding THC amount was 7 to 20 mg. However, the actual amount of cannabis taken up (i.e. the percent delivery to the respiratory tree) strongly depends on the smoking technique; it has been reported to reach approximately 50% [23]. Modern cigarettes (joint) based on intensive cannabis selection and improvement in plant cultivation contain 6 to 30% THC. Therefore, an average joint would correspond to 75 mg to 225 mg of THC! [22].

Through smoking, a 3.5% marijuana cigarette with about 900 mg plant materials can achieve plasma concentration in the range of 50 to 100 ng/ml. The maximum psychotropic effect or "high" occurs faster after smoking than by the oral route. Smoking is therefore the preferred route of cannabis administration for young users. Psychomotor function is considered to be obviously impaired above 10 ng/ml plasma THC for smoking cannabis. However in our two cases, the oral administration of cannabis produced circulating THC concentrations much lower than 10 ng/ml. We suggest several explanations for these differences. Firstly, the oral administration produces more active metabolite (11-OH-THC), which could more efficiently reach the effect site than THC. Secondly, as suggested by Chaudry [3], consuming oral cannabis may produce more potent, yet unknown psychotomimetic metabolites of THC. Thirdly, the slow absorption kinetics produces sustained plateau levels in the blood, which could influence the body and brain distribution. In a cocaine fatality, Giroud [24] found that THC and OH-THC were in higher concentration in brain than in blood.

Finally, Leweke [25] in a study including 17 healthy volunteers found also one case that suffered a two-hour episode of paranoid psychotic state following the administration of dronabinol with a lower dose than our study. Furthermore D'Souza [26] administrating intravenous THC to 22 healthy subjects in a double blind randomised clinical trial found a range of transient symptoms resembling those seen in endogenous psychosis. At last, it is important to differentiate these transient psychotic states with spontaneous resolution from the type of psychosis that persist beyond the persistence of drug in the brain, therefore probably indicating a worsening of an underlying pathologic problem.

In conclusion, doctors and users should be aware of the increasing availability of oral cannabis in "special" drinks or food as well as in medications under development. While the oral route of administration achieves only limited blood concentrations, significant psychotic reactions may occur. An increased incidence of psychotic episodes might be induced by this new trend and requires attention regarding this phenomenon in a public health perspective.

## Competing interests

The author(s) declare that they have no competing interests.

## Pre-publication history

The pre-publication history for this paper can be accessed here:



## References

[B1] Witton J, Murray RM (2004). Reefer madness revisited: cannabis and psychosis. Rev bras Psiquiatr.

[B2] Ramaekers JG, Berghaus G, Van Laar M, Drummer OH (2004). Dose related risk of motor vehicle crashes after cannabis use. Drug Alcohol Depend.

[B3] Chaudry JM, Moss HB, Bashir A, Suliman T (1991). Cannabis psychosis following bhang ingestion. British J Addiction.

[B4] Arseneault L, Cannon M, Witton J, Murray RM (2004). Causal association between cannabis and psychosis: examination of the evidence. Br J Psychiatry.

[B5] Thomas H (1996). A community survey of adverse effects of cannabis use. Drug Alcohol Depend.

[B6] Hall W (1998). Cannabis and psychosis. Drug Alcohol Rev.

[B7] Johns A (2001). Psychiatric effect of cannabis. Br J Psychiatry.

[B8] Van Os J, Bak M, Hanssen M, Bijl RV, de Graaf R, Verdoux H (2002). Cannabis use and psychosis: a longitudinal population-based study. Am J Epidemiol.

[B9] Robson P (2001). Therapeutic aspects of cannabis and cannabinoids. Br J Psychiatry.

[B10] Iversen L (2003). Cannabis and the brain. Brain.

[B11] Ames F (1958). A clinical and metabolic study of acute intoxication with cannabis sativa and its role in the model psychoses. J Ment Sci.

[B12] Talbott JA, Teague JW (1969). Marihuana psychosis: acute toxic psychosis associated with the use of cannabis derivatives. JAMA.

[B13] Tennant FS, Groesbeck CJ (1972). Psychiatric effects of hashish. Arch Gen Psychiatry.

[B14] Chopra GS, Smith JW (1974). Psychotic reactions following cannabis use in East Indians. Arch Gen Psychiatry.

[B15] Wylie AS, Scott RTA, Burnett SJ (1995). Psychosis due to "shunk". BMJ.

[B16] Emrich HM, Leweke FM, Schneider U (1997). Towards a cannabinoid hypothesis of schizophrenia: cognitive impairments due to dysregulation of the endogenous cannabinoid system. Pharmacol Biochem Behav.

[B17] Thornicroft G (1990). Cannabis and psychosis: is there epidemiological evidence for association. Br J Psychiatry.

[B18] Gruber AJ, Pope HG (1994). Cannabis psychotic disorder: does it exist?. Am J Addictions.

[B19] Thacore VR, Shukla SRP (1976). Cannabis psychosis and paranoid schizophrenia. Arch Gen Psychiatry.

[B20] Rottamburg D, Robins AH, Ben-Arie O, Teggin A, Elk R (1982). Cannabis-associated psychosis with hypomanic features. Lancet.

[B21] Imade AGT, Ebie JC (1991). A retrospective study of symptom patterns of cannabis-induced psychosis. Acta Psychiatr Scand.

[B22] World Health Organization (1997). Cannabis a health perspective and research agenda. http://whqlibdoc.who.int/hq/1997/WHO_MSA_PSA_97.4.pdf.

[B23] Agurell S, Halldin M, Lindgren JE, Ohlsson A, Widman M, Gillespie H, Hollister L (1986). Pharmacokinetics and metabolism of delta-9-tetrahydrocannabinol and other cannabinoids with emphasis on man. Pharmacol Rev.

[B24] Giroud C, Michaud K, Sporkert F, Eap C, Augsburger M, Cardinal P, Mangin P (2004). A fatal overdose of cocaine associated with coingestion of marijuana, buprenorphine, and fluoxetine. Body fluids and tissue distribution of cocaine and its metabolites determined by hydrophilic interaction chromatography-mass spectrometry (HILIC-MS). J Anal Toxicol.

[B25] Leweke FM, Schneider U, Thies M, Munte TF, Emrich HM (1999). Effects of synthetic D9-tetrahydrocannabinol on binocular depth inversion of natural and artificial objects in man. Psychopharmacology.

[B26] D'Souza DC, Perry E, MacDougall L, Ammerman Y, Cooper T, Wu YT, Braley G, Gueorguieva R, Krystal JH (2004). The psychotomimetic effects of intravenous delta-9-tetrahydrocannabinol in healthy individuals: implications for psychosis. Neuropsychopharmacology.

